# Atopic dermatitis in early life and pain at 10 years of age: An exploratory study

**DOI:** 10.1007/s00431-024-05439-0

**Published:** 2024-02-24

**Authors:** Vanessa Gorito, Maria Brandão, Inês Azevedo, André Moreira, Raquel Lucas

**Affiliations:** 1 EPIUnit – Instituto de Saúde Pública e Laboratório para a Investigação Integrativa e Translacional em Saúde Populacional (ITR), Universidade do Porto, Rua das Taipas 135, 050-600 Porto, Portugal; 2 Serviço de Pediatria e Neonatologia, Centro Hospitalar de Trás-os-Montes e Alto Douro, Vila Real, Portugal; 3 Departamento de Ginecologia e Obstetrícia, Faculdade de Medicina, Universidade do Porto, Porto, Portugal; 4 Serviço de Pediatria Médica, UAG da Mulher e Criança, Centro Hospitalar e Universitário de São João, Porto, Portugal; 5 Serviço de Imunoalergologia, Centro Hospitalar Universitário São João, Porto, Portugal; 6 Unidade de Imunologia Básica e Clínica, Departamento de Patologia, Faculdade de Medicina, Universidade do Porto, Porto, Portugal; 7 Instituto de Ciências Biomédicas Abel Salazar, Universidade do Porto, Porto, Portugal

**Keywords:** Atopy, Atopic dermatitis, Sensitization, Pediatric pain, Cohort study, Child health

## Abstract

Pain is a distinctive burden in atopic dermatitis and recognized as an important and highly prevalent symptom. It is unknown if the presence of atopic disease may sensitize children to adverse pain profiles in the long term. We aimed to assess the impact of early-life atopic dermatitis-like symptoms on pain at 10 years of age. We used data from 1302 and 874 participants of the Generation XXI birth cohort evaluated at 6 and 15 months, respectively, and 10 years. Atopy-like symptoms since birth, including atopic dermatitis, were collected at ages 6 and 15 months by interviewing parents. Pain history in the last 3 months at age 10 was collected from parents and children using structured questionnaires. We computed relative risks (RR) and respective 95% confidence intervals of pain features at age 10 according to each atopic-like symptom at 6 and 15 months. Children whose parents reported atopic dermatitis-like symptoms at 6 months and at 15 months had higher risk of reporting any pain (RR 1.75 [1.15–2.66]) and multisite pain, respectively (RR 1.67 [1.18–2.37]) at 10 years of age.

*Conclusion*: Atopic dermatitis symptoms in early life were associated with a higher risk of pain at age 10, suggesting that potential for sensitization during the first decade of life and highlighting the importance of improving the health care of children with atopic dermatitis is worth investigating.

**Table:** 

**What is Known:** *• Atopic disorders have been associated with many non-atopic comorbidities, including chronic pain.* *• Pain and atopic dermatitis share common inflammatory pathways. Inflammation, injury to the skin from scratching, fissures, and intolerance to irritants related to atopic dermatitis can cause pain.*	
**What is New:** *• Atopic dermatitis in early life is linked to an increased likelihood of experiencing pain at the age of 10, which suggests that exploring the potential for sensitization is a worthwhile area of investigation.* *• Our proof-of-concept study highlights the potential benefit of studying management targets and improving itching and relieving skin pain as quickly as possible, avoiding potential long-term consequences of the sensitization process.*

**Supplementary Information:**

The online version contains supplementary material available at 10.1007/s00431-024-05439-0.

## Introduction

Atopic disorders have been associated with many non-atopic comorbidities, including chronic pain [1, 2]. It is thought that nociceptive pain events in the first years of life can partly trigger amplified pain syndromes [3]. In recent years, there has been a growing focus on the burden of pain in patients with atopic dermatitis, one of the most frequent chronic skin diseases of childhood^1,2^ that affects around 20% of children in high-income countries [1, 4].

Atopic dermatitis has been described as inducing significant somatic suffering and psychological disturbance (sleep disorders, depression, anxiety) with massive economic burden [5]. Epidermal barrier disruptions and skin inflammation are cornerstones of atopic dermatitis pathophysiological processes. A consequence of the interaction between a compromised barrier and a type 2 immune environment is the promotion of percutaneous allergic sensitization [6]. Although itch is the most prominent symptom, the pain has recently been considered a nonnegligible burden in atopic dermatitis [5, 6]. Inflammation, injury to the skin from scratching, fissures, and intolerance to irritants related to atopic dermatitis can cause pain that ranges in intensity from aggravating to agonizing. Huet et al. in an investigation published recently revealed that among adult participants with atopic dermatitis, more than a half dealt with pain issues at least once a week and five percent had pain daily [7]. Silverberg et al. found that about half of the adults and adolescents with atopic dermatitis in the study experienced pain [8].

Pain and atopic dermatitis share common inflammatory pathways. IL-1β has been implicated in painful and inflammatory processes, namely promoting hyperalgesia [9]. Elevating the inflammatory cytokines IL-1β, IL-6, and TNF-α in fibromyalgia suggests an inflammatory component in the induction of widespread pain [2]. Besides, serum levels of IL-8, TNF-α, and IL-6 may be important markers of disease severity and for the follow-up of children with atopic disorders [10].

We hypothesized that atopic dermatitis may have a prospective influence on pain experiences in children. Also, in large-scale population-based studies, without a clinical evaluation, atopic-like symptoms can be used as a proxy of atopy, even if not optimally specific. Therefore, we used prospective data from a population-based cohort to study the association between early-life atopic dermatitis, among other atopic-like symptoms, and pain experiences in school-age children.

## Materials and methods

### The Generation XXI (G21) cohort study

Generation XXI is a birth cohort study that enrolled 8647 newborns, in 2005–2006, in the five public maternities covering the metropolitan area of Porto, Portugal. A baseline evaluation was done during the hospital stay (24–72 h after the delivery) with a 91.4% participation proportion. Consecutive subsamples of babies were examined at 6 and 15 months and the whole cohort was invited to participate in evaluations at 4, 7 and 10 years. In each evaluation wave, questionnaires collected extensive information, including demographic and socioeconomic characteristics, clinical history, common childhood symptoms, and pain experiences [11, 12].

We present the sociodemographic and clinical characteristics of our sample on Table 1.

**Table 1:** Sociodemographic and clinical characteristics of G21 participants who were included in this study

	**Evaluated at 6 months and 10 years** **(*N* = 1302)**	**Evaluated at 15 months and 10 years** **(*N* = 874)**
**Sex at birth**		
Female	638 (49%)	442 (51%)
Male	664 (51%)	432 (49%)
**Maternal age at the child’s birth (years)**		
< 26	274 (21%)	171 (20%)
26–35	859 (66%)	583 (67%)
≥ 36	169 (13%)	120 (13%)
**Maternal education**		
< 10th grade	520 (40%)	391 (45%)
10th–12th grade	379 (29%)	240 (27%)
> 12th grade	403 (31%)	243 (28%)
**Monthly household income (EUR)**		
Under 1000	408 (31%)	324 (37%)
1000 to 2000	650 (50%)	413 (47%)
Above 2000	244 (19%)	137 (16%)
**Type of delivery**		
Eutocic	645 (50%)	416 (48%)
Forceps	15 (1.2%)	9 (1.0%)
Vacuum	195 (15%)	122 (14%)
Cesarean	447 (34%)	327 (37%)
**Gestational age at child’s birth**		
< 37 weeks	115 (8.8%)	75 (8.6%)
≥ 37 weeks	1187 (91%)	799 (91%)
**Low birth weight (< 2500 g)**		
Yes	108 (8.3%)	79 (9.0%)
No	1194 (92%)	795 (91%)
**Child neonatal resuscitation**^a^		
Yes	117 (8.9%)	85 (9.7%)
No	1185 (91%)	789 (90%)
**Admission to neonatal intensive care unit**		
Yes	94 (7.2%)	69 (7.9%)
No	1208 (93%)	805 (92%)

^a^Defined as the set of interventions at the time of birth to support the establishment of breathing and circulation

### Data collection

#### Early life atopic-like symptoms at 6 months and 15 months

At 6 and 15 months, subsamples of 1555 and 1043 babies were randomly selected from the whole cohort and examined, respectively. We used standardized questionnaires and asked parents to report atopic-like symptoms experienced by the child since birth, as described below. At 6 months, personal history of food allergy/intolerance-like symptoms, atopic dermatitis-like symptoms and wheezing-like symptoms were evaluated through the following questions: “Did your baby ever have an episode of wheezing, whistling or rattling in the chest?”, “Did your baby ever have an episode of food allergy/intolerance?”, and “Did your baby ever have an episode of itchy skin changes/eczema?”.

At 15 months, the child’s history of food allergy-like symptoms, atopic dermatitis-like symptoms and wheezing-like symptoms were repeated, and an additional question was included: “Did your baby ever have “persistent symptoms such as sneezing, nasal itching, rhinorrhea or nasal congestion without a cold/flu?”.

#### Pain at 10 years of age

In the 10 years of evaluation, about 74% of all the cohort had been evaluated; 6397 parents and 4752 children answered a structured questionnaire focused on the child’s health and surrounding context, including the child’s experience of pain. The whole cohort was invited to participate, including participants with diagnosed chronic conditions reported by parents, namely atopic disorders, independently of flare symptoms at 10 years evaluation.

From all cohort, we used information provided for 83% of the subsample evaluated at 6 months and 84% of the subsample evaluated at 15 months who had complete information about pain at 10 years of follow-up.

To evaluate the pain experience, a Portuguese version of the Luebeck pain screening questionnaire was applied [13]. The first (screening) question was, “Did your child complain of pain in the last 3 months?” If the answer was affirmative, the parent was asked to select the anatomical sites where the child felt pain and indicate the principal pain site according to parental assessment [14]. We considered multisite pain to be present if two or more anatomical regions (among head, chest, abdomen, pelvis, back, upper limb, lower limb, throat, ear, teeth or other) were selected and chronic pain if the child had at least one recurrent pain with a duration over 3 months. Pain intensity was assessed using the original six-point Faces Pain Scale (FPS). Intense pain was considered if FPS4 or higher was selected [15].

Using a brief questionnaire, children also answered questions about their pain experience in the previous week: the presence of some pain, pain intensity (intense pain was considered if FPS4 or higher was selected) and the number of pain sites, using an age-appropriate body chart. Multisite pain was considered present if two or more anatomical regions were selected (among head, chest, abdomen, back, hip, shoulder, arm, hand, leg, foot or other) [14].

### Statistical analysis

We describe variable distributions using proportions by categories and computed risks of pain features at age ten reported by parents and children according to the presence of each atopic-like symptom at 6 and 15 months, using data of participants with complete information on all relevant variables.

We compared sociodemographic and clinical characteristics at the baseline of participants that fulfilled the inclusion criteria with all other participants of the G21 cohort to assess losses to follow-up. The following variables were collected from clinical records at childbirth: sex at birth (female or male); maternal age at birth (< 26 years, 26–35 years, ≥ 36 years); type of delivery (eutocic, forceps, vacuum or cesarean section); gestational age at birth (< 37 weeks; ≥ 37 weeks); low birth weight (below 2500 g vs 2500 g or above); need for resuscitation at birth (yes or no); admission to a neonatal intensive care unit (yes or no). Maternal education (< 10th grade, 10th–12th grade, > 12th grade) and household monthly income (under EUR 1000, EUR 1000 to 2000, or above EUR 2000) were collected by questioning the mother at birth using standardized questionnaires applied by trained interviewers up to 72 h after delivery.

Non-eligible participants had similar sex distribution, maternal education, household income, type of delivery and birth weight proportions. However non-eligible participants are more frequently premature (9.1%) and admitted in the Neonatal Intensive Care Unit (8.9%) (Table 1 — Supplementary information).

As atopic dermatitis may cause a predisposition to additional atopic illnesses in pediatric age and the presence of these illnesses correlates with poor disease control, we created a dichotomous variable “*multiple atopic symptoms*” based on the presence/absence of two or more atopic-like symptoms at 6 months or 15 months [31, 32]. The magnitude of the associations between reported symptoms and pain features was estimated using relative risks (RR) and asymptotic confidence intervals (95% CI). We adjusted associations for potential confounding factors, through a multivariable regression model including as co-variates sex, maternal education, household income, type of delivery and gestational age at birth.

All statistical analyses were performed using SPSS version 27 and we used a significance threshold of *p* = 0.05.

## Results

### Cohort characterization

**Fig. 1:**
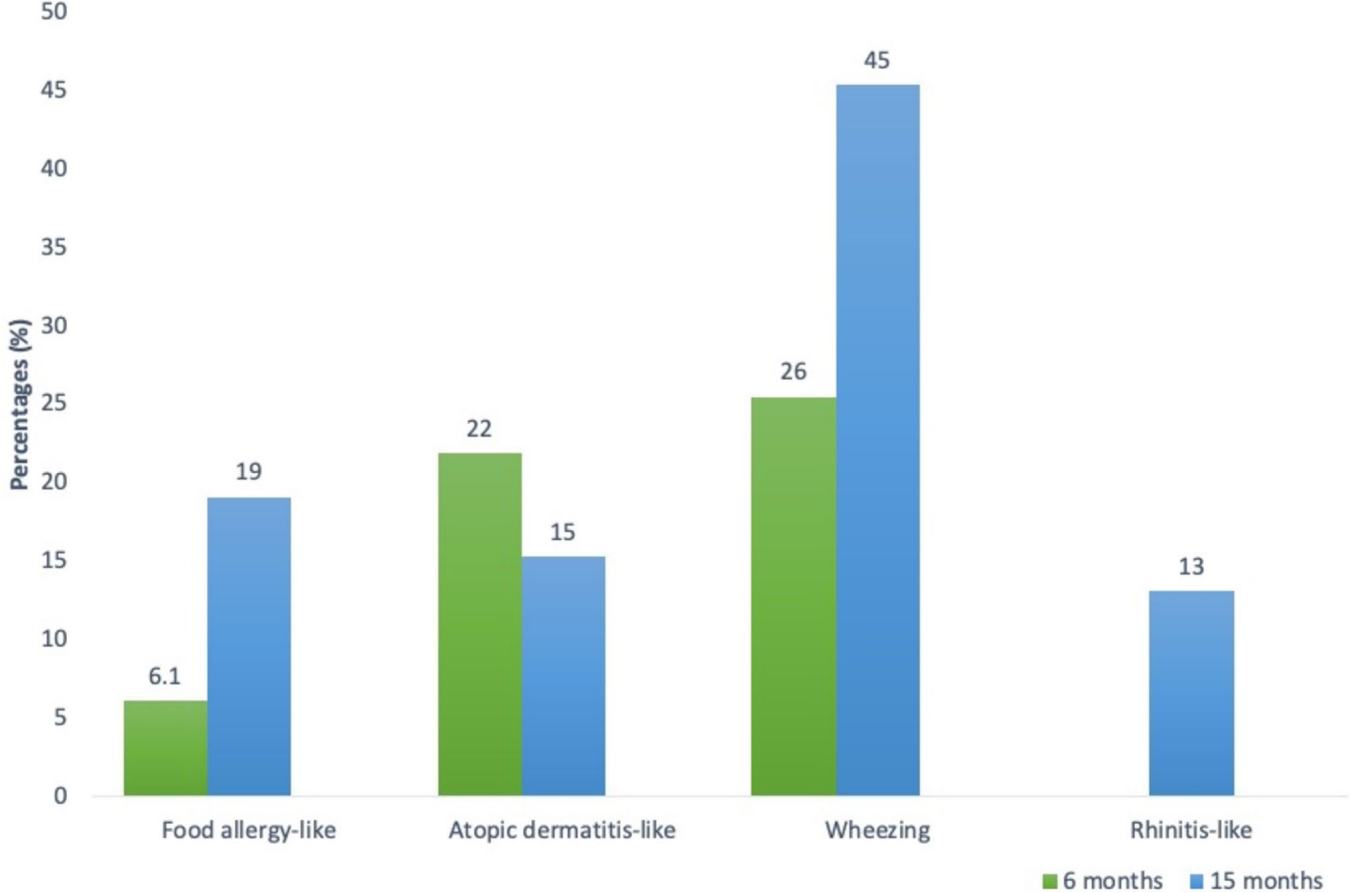
Frequencies (%) of atopic-like symptoms in evaluations at 6 months and 15 months

Of the 1555 children evaluated at 6 months of age, as reported by parents, 396 (26%) had at least a wheezing episode, 340 (22%) had an atopic dermatitis-like episode, and 129 (8.1%) had food allergy-like symptom. Of the 1043 children evaluated at 15 months of age, parents reported wheezing episodes in 474 (45%), atopic dermatitis-like symptoms in 160 (15%), food allergy-like symptoms in 199 children (19%), and rhinitis-like symptoms in 131 (13%) (Fig. 1).

**Fig. 2:**
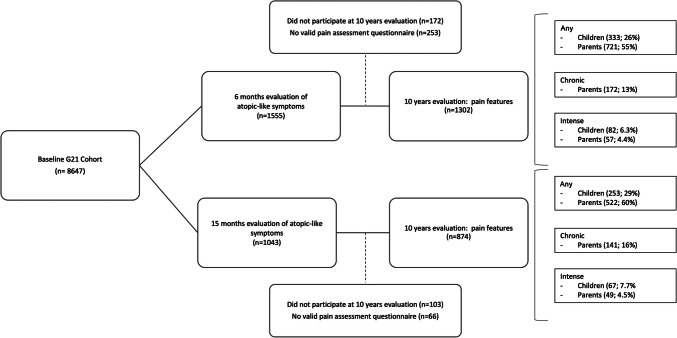
Characterization of the pain experience at 10 years, considering subsamples of children consecutively selected from the whole cohort and evaluated at 6 months and 15 months for presence of atopy-like symptoms

At 10 years of age, 1302 of the 1555 children evaluated at 6 months and 874 of the 1043 children evaluated at 15 months had both parent and child reports of pain. Among those who were evaluated at 6 months and 10 years of age, 333 (26%) children reported having some pain, 82 (6.3%) intense pain, and 98 (7.5%) multisite pain in the week before the evaluation. According to parents, 721 (55%) of those children had some pain, 172 (13%) chronic pain, 57 (4.4%) intense pain and 307 (24%) multisite pain in the three months preceding the evaluation (Fig. 2). Of the subsample evaluated at 15 months and 10 years of age, 253 (29%) children reported having some pain, 67 (7.7%) intense pain, and 49 (5.6%) multisite pain in the week before the evaluation. According to parents, 522 (60%) children had some pain, 141(16%) had chronic pain, 49 (5.6%) had intense pain and 208 (24%) had multisite pain during the previous three months (Fig. 2).

### Atopic dermatitis-like symptoms and pain

Considering the parental report of pain, children with reported atopic dermatitis-like episodes at 15 months had higher risk of multisite pain at age 10 (42% vs 30%; RR 1.67 [1.18–2.37]). Atopic dermatitis-like episodes at 15 months were not associated to the presence of any pain (62% vs 59%; RR 1.16 [0.86–1.55]), intense (26% vs 23%; RR 1.16 [0.59–2.28]), and chronic pain reported by parents (67% vs 64%; RR 1.13 [0.62–2.06]). No associations were found between atopic dermatitis at 6 months and reported pain by parents at 10 years (Table 2).

**Table 2:** Association between diagnosed atopic-like symptoms in children evaluation at 6 months and 15 months and presence of any chronic, intense and multisite pain according to parents' report at 10 years

	**Any pain**			**Chronic pain**			**Intense pain**			**Multisite pain**		
***N* = 1302** 6-months	%	RR [95% CI]^b^	RR [95% CI] adjusted^a^	%	RR [95% CI]^b^	RR [95% CI] adjusted^a^	%	RR [95% CI]^b^	RR [95% CI] adjusted^a^	%	RR [95% CI]^b^	RR [95% CI] adjusted^a^
Food allergy-like												
-Yes -No	61 59	1.08 [0.72–1.63]	0.68 [0.28–1.62]	68 62	1.29 [0.58–2.86]	1.49 [0.61–3.56]	27 22	1.32 [0.55–3.13]	1.10 [0.42–3.35]	25 26	0.94 [0.58–1.50]	0.76 [0.42–1.39]
Atopic dermatitis-like												
-Yes -No	57 59	0.91 [0.61–1.35]	0.96 [0.41–2.24]	64 63	1.06 [0.47–2.41]	0.92 [0.41–2.24]	21 21	1.00 [0.38–2.61]	1.02 [0.35–1.44]	28 26	1.14 [0.70–1.85]	1.22 [0.62–2.44]
Wheezing												
-Yes -No	57 60	0.88 [0.68–1.14]	0.71 [0.38–1.30]	67 61	1.25 [0.70–2.22]	0.98 [0.54–1.75]	21 20	1.03 [0.51–2.74]	1.00 [0.48–2.09]	26 25	1.05 [0.78–1.42]	0.63 [0.42–1.02]
Atopic factors												
-0–1 -≥ 2	58 60	1.06 [0.72–1.57]	1.02 [0.61–1.37]	13 19	1.53 [0.93–2.51]	1.01 [0.57–1.31]	21 22	1.08 [0.43–2.67]	0.99 [0.54–1.75]	25 31	1.38 [0.91–2.10]	1.12 [0.64–1.75]

***N* = 874** 15-months	%	RR [95% CI]^b^	RR [95% CI] adjusted^a^	%	RR [95% CI]^b^	RR [95% CI] adjusted^a^	%	RR [95% CI]^b^	RR [95% CI] adjusted^a^	%	RR [95% CI]^b^	RR [95% CI] adjusted^a^
Food allergy-like												
-Yes -No	68 58	1.59 [1.12–2.27]*	1.70 [1.14–2.54]*	65 53	1.1 [0.58–2.08]	1.67 [0.36–7.76]	32 21	1.79 [0.88–3.64]	0.49 [0.22–1.13]	31 34	0.96 [0.63–1.46]	0.44 [0.06–3.44]
Atopic dermatitis-like												
-Yes -No	62 59	1.16 [0.86–1.55]	1.19 [0.59–2.40]	67 65	1.13 [0.62–2.06]	1.01 [0.39–2.64]	26 23	1.16 [0.59–2.28]	0.56 [0.22–1.13]	42 30	1.67 [1.18–2.37]*	1.22 [1.15–2.43]*
Wheezing												
-Yes -No	67 54	1.64 [1.25–2.17]**	1.56 [1.15–2.11]**	64 63	1.06 [0.61–1.85]	1.07 [0.56–2.06]	22 24	0.89 [0.47–1.70]	0.85 [0.41–1.78]	38 30	1.43 [1.02–2.01]*	1.58 [1.17–2.15]**
Rhinitis-like												
-Yes -No	66 58	1.38 [0.98–1.94]	0.75 [0.38–2.4]	70 62	1.41 [0.75–2.64]	0.60 [0.27–1.29]	32 20	1.91 [0.96–3.79]	0.59 [0.27–1.23]	31 34	0.93 [0.63–1.40]	0.35 [0.02–2.33]
Atopic factors												
-0–1 -≥ 2	57 60	1.67 [0.36–7.76]	1.27 [0.43–4.21]	14 23	1.82 [0.21–15.76]	1.37 [0.53–5.11]	14 33	1.50 [0.23–1.83]	1.07 [0.53–1.71]	45 31	0.15 [0.02–1.51]	0.37 [0.12–2.13]

*RR* Relative Risk, *CI* Confidence Interval

*p < 0.05; **p < 0.001

^a^Model adjusted for: sex, maternal education, household income, type of delivery and gestational age

^b^Reporting pain features

Considering the children report of pain, atopic dermatitis-like symptoms at 6 months had higher risk of reporting any pain in the previous week at 10 years of age than those without atopic dermatitis (37% vs 25%; RR 1.75 [1.15–2.66]); but not intense pain (26% vs 24%; RR 1.08 [0.50–2.33] or multisite pain (8.30% vs 10%; RR 0.80 [0.35–1.89). Atopic dermatitis-like at 15 months was not associated with reports of intense or multisite pain at 10 years by children (Table 3).

**Table 3:** Association between diagnosed atopic-like symptoms in children evaluation at 6 months and 15 months and presence of any chronic, intense and multisite pain according to children´s report at 10 years

	**Any pain**			**Intense pain**			**Multisite pain**		
***N* = 1302** 6-months	%	RR [95% CI]^b^	RR [95% CI] adjusted^a^	%	RR [95% CI]^b^	RR [95% CI] adjusted^a^	%	RR [95% CI]^b^	RR [95% CI] adjusted^a^
Food allergy-like									
-Yes -No	29 25	1.21 [0.78–1.88]	0.74 [0.47–1.16]	29 24	1.22 [0.58–2.99]	0.92 [0.38–-2.21]	11 10	1.12 [0.56–2.23]	0.22 [0.09–0.59]
Atopic dermatitis-like									
-Yes -No	37 25	1.75 [1.15–2.66]**	1.75 [1.14–2.69]*	26 24	1.08 [0.50–2.33]	0.91 [0.41–2.01]	8.3 10	0.80 [0.35–1.89]	1.13 [0.41–3.10]
Wheezing									
-Yes -No	29 24	1.25 [0.94–1.67]	1.18 [0.67–2.24]	26 21	1.32 [0.75–2.33]	1.18 [0.63–2.24]	10 10	1.01 [0.61–1.66]	0.48 [0.19–1.21]
Atopic factors									
-0–1 -≥ 2	25 30	1.29 [0.84–1.96]	1.14 [0.77–1.76]	22 23	1.06 [0.46–2.46]	1.02 [0.29–2.47]	10 7.5	0.31 [0.31–1.55]	0.58 [0.29–1.41]

***N* = 874** 15-months	%	RR [95% CI]^b^	RR [95% CI] adjusted^a^	%	RR [95% CI]^b^	RR [95% CI] adjusted^a^	%	RR [95% CI]^b^	RR [95% CI] adjusted^a^
Food allergy-like									
-Yes -No	27 29	0.91 [0.63–1.33]	1.11 [0.72–1.69]	30 25	1.24 [0.62–2.50]	0.68 [0.29–1.60]	9.9 7.6	1.34 [0.67–2.65]	1.84 [0.40–8.22]
Atopic dermatitis-like									
-Yes -No	28 29	0.94 [0.69–1.29]	1.03 [0.32–3.33]	28 23	0.75 [0.40–1.39]	0.68 [0.21–2.17]	6.9 9.0	0.85 [0.40–1.42]	0.37 [0.08–1.70]
Wheezing									
-Yes -No	31 28	1.17 [0.87–1.56]	0.83 [0.60–1.15]	24 28	0.81 [0.46–1.44]	1.48 [0.76–2.89]	8.2 8.1	1.01 [0.56–1.80]	0.63 [0.24–1.63]
Rhinitis-like									
-Yes -No	31 229	1.12 [0.78–1.60]	0.88 [0.59–1.31]	30 25	1.27 [0.66–2.45]	1.22 [0.56–2.64]	7.5 7.9	0.94 [0.46–1.95]	0.97 [0.31–3.02]
Atopic factors									
-0–1 -≥ 2	29 29	1.02 [0.19–5.47]	1.00 [0.17–3.16]	12 31	1.45 [0.19–1.77]	1.12 [0.19–1.47]	0.9 8.1	1.09 [0.03–1.15]	1.07 [0.10–1.25]

*RR* Relative Risk, *CI* Confidence Interval

*p < 0.05; **p < 0.001

^a^model adjusted for: sex, maternal education, household income, type of delivery and gestational age

^b^reporting pain features

We adjusted the relative risks for potential confounding factors, through a multivariable regression model including sex, maternal education, household income, type of delivery and gestational age. No major differences were found in findings after adjustment for both parents (Table 2) and children (Table 3) reports of pain.

### Other atopic dermatitis-like symptoms and pain

Considering parental report of pain, wheezing episodes and food-allergy symptoms at 6 months had weak or no associations with pain, chronic pain, multisite and intense pain at 10 years. Children with food-allergy-like symptoms at 15 months had a higher risk of reported pain at age 10 (Table 2). Presence of rhinitis-like symptoms at 15 months was borderline associated with any and intense pain, but not with chronic or multisite pain at 10 years. There was no clear association between the presence of two or more atopy-like symptoms at 6 and 15 months and pain at 10 years of age (Table 2).

As for child report of pain, children with wheezing episodes at 6 months had slightly higher risk of any pain, but not intense or multisite pain at age 10. No clear associations were found between food allergy symptoms at 6 months and child reports of any pain or multisite pain at age 10 (Table 3). Food allergy-like symptoms at 15 months were not associated with reports of intense or multisite pain at ten years. There were no differences in the risk of any type of pain between children with and without wheezing episodes at 15 months. No clear associations were found between rhinitis-like symptoms and any, intense, or multisite pain (Table 3). We found no clear association between the presence of multiple atopic symptoms at 6 months and the presence of intense or multisite pain. Reports of any pain by children were slightly more common when two or more atopic symptoms were present at 6 months. The presence of two or more atopic symptoms at 15 months was not associated with the risk of any pain, intense pain, or multisite pain (Table 3).

We adjusted the relative risks for potential confounding factors, through a multinomial regression model including sex, maternal education, household income, type of delivery and gestational age. No differences were found in calculated risks after this adjustment for both parents (Table 2) and children (Table 3) reports of pain.

## Discussion

Our prospective cohort study partly confirms our hypothesis, given that atopic dermatitis seemed to increase the risk of pain reported by children, as well as the report of multisite pain according to parents. Besides the increased risk of pain associated with atopic dermatitis in early life, food allergy-like symptoms and wheezing episodes at 15 months were also associated with future pain reported by parents, but those results were less robust.

Atopic dermatitis can negatively affect sensorimotor and cognitive development, behavior, pain responses, pharmacological requirements, and long-term health status of the children [15, 16]. Multisite pain is one of the most consensual indicators of adverse pain experiences in pediatric ages [17] and associations with atopy-like symptoms were slightly more robust with multisite and intense pain than with chronic pain.

Atopic skin with an impaired barrier, neurogenic inflammation mediators, and peripheral and central sensitization of pain processing pathways may possibly explain nociplastic pain mechanisms in atopic dermatitis [18]. Skin pain is increasingly recognized as an impactful symptom in atopic atopic dermatitis because of its association with patient discomfort, disease burden, and reduced quality of life [6, 7]. Although the nature of skin pain in atopic atopic dermatitis has not been systematically studied and is therefore not well understood, patients report soreness, discomfort, and tenderness that may reflect peripheral and central pain sensitization [8, 18]. Research suggests that peripheral inflammatory mediators contribute to pain [19, 20]. For instance, Cathepsin S activates T CD4+ cells, resulting in neuropathic pain [19] and IL‐33 has been demonstrated to boost inflammation and pain [20]. Both inflammatory mediators are reported to be overexpressed in atopic dermatitis [20]. Nerve growth factor (NGF) is implicated in chronic pain conditions by primary pain afferents and the level of NGF was confirmed to be increased in atopic dermatitis patients [21].

Considering peripheral sensitization to pain, G protein‐coupled receptors (GPCRs) and transient receptor potential (TRP) channels, contribute to skin sensation [22]. TRP vanilloid 1 (TRPV1), TRP ankyrin 1 (TRPA1), and transient receptor melastatin member 8 (TRPM8) are detected at high levels in atopic lesions [23]. Activation of TRPV1 results in increased levels of proinflammatory neuropeptide substance P, accelerating neurogenic inflammation [24]. Mas‐related GPCRs (Mrgprs) are expressed by primary sensory neurons for peripheral skin sensations [25]. Mas‐related GPCR D (MrgprD) is likely to be involved in neuropathic pain mechanisms as a nociceptor and mediate pain signaling [26].

Considering central sensitization of pain, the endothelin‐1/endothelin receptor type B (ET‐1/EDNRB) pathway was implicated in an atopic mouse model to explain the correlation between neuropathic pain and allergic inflammation, as also supported by an enhanced level of ET‐1 in atopic patients [27]. The activation of spinal microglia boosts the expression of cytokines including IL‐1β and TNF‐α and brain‐derived neurotrophic factor [28]. Besides, chronic pain‐associated brain areas are activated in the anterior cingulate cortex, insula, and dorsolateral prefrontal cortex of the brain in atopic dermatitis patients, as observed by brain imaging [29].

The sensitization process that can lead to the perception of non-nociceptive stimuli as painful, as well as exaggerated (prolonged and generalized) perception of pain from noxious stimuli [6, 7] seems to be even more intense in early life, which justifies examining the associations between atopic dermatitis in early life and the occurrence of pain at age 10 years.

### Strengths of the study

Our study examined atopy-like symptoms in early life as potential contributors for pain experiences at 10 years using a large population-based sample of children during their childhood. As far as we know this is the first prospective study of this association in a population-based sample of children, evaluated prospectively in different stages and selected independently of symptoms or healthcare seeking behavior.

We found associations between atopic dermatitis and both children and parental pain reports, even after adjustment for sociodemographic and perinatal confounders (sex, maternal education, household income, type of delivery and gestational age) contributing to making our hypothesis more plausible. Although children are able to report their pain at 10 years, and should be always asked about their pain, parents give valuable information to complement children’s report. Parents are likely to notice any discomfort or change in children's behavior related to atopy-like symptoms and pain complaints, although our previous investigation in this cohort suggests that parental report is more useful to rule-in than to rule-out the child’s pain [30]. We consider the use of both records (children and parents) a major strength of this study.

### Limitations of the data

Our study may be limited because it is based on reports, without doctors’ information/clinical validation of the diagnosis of atopy and without a clinical attribution for pain reports, e.g. pain at age 10 could be directly related to an atopic disease flare and not a result of cumulative sensitization. We measured atopic-like symptoms in the first 15 months of life as reported by parents and not the prevalence of established/diagnosed atopic diseases, which would likely be lower if validated using clinical records. For instance, food allergy-like symptoms are often confused with food intolerances or isolated symptoms, and many respiratory infections can cause wheezing. Atopic dermatitis can also be mistaken for a nonspecific rash that may appear in early childhood. We conducted our study by selecting sub-samples of children with early-life atopic events from a population cohort and our results may be influenced by bias related to non-participation and/or attrition. It should also be noted that parents who report atopic-like symptoms may be more likely to report pain experiences due to being more health-conscious or more vulnerable to physical complaints, which could induce an association between atopy and pain due to surveillance bias.

### Future approaches

There are different scales/questionnaires to assess the impact of atopic dermatitis on patients’ lives [33]. In the near future, it would be advantageous to screen the pain complaints/discomfort routinely in appointments to improve the prevention measures and to diagnose and to treat flares earlier. The current evidence and advances for the potential treatment of atopic dermatitis have been studied by targeting specific interleukin (p.e IL1, IL33), common in both inflammatory pathways pain in atopic dermatitis [34]. Pharmacological treatment targeting these specific interleukines (p.e canakinumab) may be a potential treatment in atopic dermatitis especially associated with pain [34]. Nevertheless, ours is an exploratory study and a lot remains to be clarified regarding the complex early life influences on chronic pain in the general population, as seen, e.g. in a recent cohort study where preterm birth was associated with less severe spinal pain in pre-adolescence among girls [35].

## Conclusion

In our population-based study, atopic dermatitis symptoms in early life were associated with a higher risk of pain at age 10, which could be a starting point to bring together the study of atopic dermatitis and pain sensitization in children. We highlight the potential benefit of studying strategies to address itching and skin pain to avoid pain sensitization processes that may contribute to long-term chronic pain phenotypes and to improve the care of children with atopic dermatitis.

### Supplementary information

Below is the link to the electronic supplementary material.

Supplementary file1 (DOCX 24 KB)

## Data Availability

Data are made available upon request, after submission of the scientific proposal to the executive committee of the cohort (info@geracao21.com).
