# Supplementary material for: Atopic dermatitis in early life and pain at 10 years of age: An exploratory study
Source: Eur J Pediatr. 2024 Feb 24;183(5):2239–49. doi: 10.1007/s00431-024-05439-0 (PMC11035428; doi:10.1007/s00431-024-05439-0)
Supplement: Supplementary file 1 — Supplementary file1 (DOCX 24 KB) [file 431_2024_5439_MOESM1_ESM.docx]

Table 1. Sociodemographic and clinical characteristics of G21 non-eligible participants in this study

|  | **Non-eligible participants evaluated at 6 months and 10 years**  **(N=253)** | |  | **Non-eligible participants evaluated at 15 months and 10 years**  **(N=169)** | |
| --- | --- | --- | --- | --- | --- |
| **Sex at birth** | |  |  |  | |
| Female | | 124 (49%) |  | 86 (51%) | |
| Male | | 129 (51%) |  | 83 (49%) | |
| **Maternal age at the child’s birth (years)** | |  |  |  | |
| < 26 | | 53 (21%) |  | 34 (20%) | |
| 26-35 | | 165 (65%) |  | 113 (67%) | |
| ≥ 36 | | 35 (14%) |  | 22 (13%) | |
| **Maternal education** | |  |  |  | |
| < 10th grade | | 102 (40%) |  | 77 (45%) | |
| 10th - 12th grade | | 73 (29%) |  | 47 (28%) | |
| > 12th grade | | 78 (31%) |  | 45 (27%) | |
| **Monthly household income (EUR)** | |  |  |  | |
| Under 1000 | | 78 (31%) |  | 62 (37%) | |
| 1000 to 2000 | | 127 (50%) |  | 81 (48%) | |
| Above 2000 | | 48 (19%) |  | 26 (15%) | |
| **Type of delivery** | |  |  |  |  |
| Eutocic | | 127 (49%) |  | 80 (47%) |  |
| Forceps | | 4 (1.6%) |  | 2 (1.2%) |  |
| Vacuum | | 36 (14%) |  | 24 (14%) |  |
| Cesarean | | 86 (34%) |  | 63 (37%) |  |
| **Gestational age at child’s birth** | |  |  |  |  |
| < 37 weeks | | 23 (9.1%) |  | 13 (8.3%) |  |
| ≥ 37 weeks | | 230 (91%) |  | 156 (92%) |  |
| **Low birth weight (<2500g)** | |  |  |  |  |
| Yes | | 21 (8.3%) |  | 15 (8.9%) |  |
| No | | 232 (92%) |  | 154 (91%) |  |
| **Child neonatal resuscitation ^a^** | |  |  |  |  |
| Yes | | 22 (8.7%) |  | 17 (10%) |  |
| No | | 231 (91%) |  | 152 (90%) |  |
| **Admission to neonatal intensive care unit**  Yes | | 18 (7.1%) |  | 15 (8.9%) |  |
| No | 235 (93%) | |  | 154 (91%) |  |

^a^ defined as the set of interventions at the time of birth to support the establishment of breathing and circulatio
